# Microbial Assessment of Reclosable Single-Day Use Ophtioles Containing Autologous Serum Eye Drops

**DOI:** 10.3390/bioengineering13040417

**Published:** 2026-04-02

**Authors:** Frank Blaser, Daniel Barthelmes, Germaine Korner, Julia Bugajska, Oliver Nolte, Sandrine Anne Zweifel, Sadiq Said, Schneider Jana, Isabelle Meneau, Anahita Bajka

**Affiliations:** 1 Department of Ophthalmology, University Hospital Zurich, University Zurich, 8091 Zurich, Switzerland; 2 Bioexam AG, 6006 Lucerne, Switzerland; 3 Department of Immunology, University Hospital Zurich, University Zurich, 8091 Zurich, Switzerland

**Keywords:** autologous serum eye drops, blood product, contamination, sterility

## Abstract

**Background:** Autologous serum eye drops (ASEDs) are produced without preservatives and need to be stored at 2–8 °C while in use. This study aims to analyze the behavior of specific bacteria and fungi in the case of contamination during the usage of single-day use ophtioles containing ASED. **Methods:** This is a prospective experimental study conducted at the Department of Ophthalmology, University Hospital of Zurich (USZ), and a regulatory-licensed, independent, external laboratory. The laboratory performed microbial testing on the ASEDs with 11 microorganisms in 100% concentration or 50% diluted using the original 2.5 mL ASED ophtioles provided by the Eye Bank of the Department of Ophthalmology, USZ. Storage took place at 2–8 °C and 20–25 °C. The acceptance criterion was the absence of microbial growth between opening (T0), 24 h afterwards (T24), and 48 h afterwards (T48). **Results:** The acceptance criteria were met for all microorganisms for 2–8 °C. For seven microorganisms, the acceptance criteria were met for 2–8 °C and 20–25 °C. For four microorganisms, the acceptance criteria were only met for 2–8 °C. **Conclusions:** No relevant growth was observed in any of the test strains from T0 to T24 and T48 at 2–8 °C, demonstrating the microbiological safety of reclosable single-day use ophtioles containing unpreserved ASEDs when stored at 2–8 °C.

## 1. Introduction

Over recent decades, autologous serum eye drops (ASEDs) have been established worldwide as a second-line treatment modality for moderate-to-severe ocular surface disease that fails to respond to conventional lubricating eye drops [1,2,3,4,5,6,7,8,9]. The rationale for using ASEDs is the biochemical similarity of autologous serum and tear fluid, including the presence of growth factors and proteins essential for epithelial growth [2,10,11]. Several studies have reported symptomatic and objective improvements in patients using ASEDs, with a favorable safety profile [4,7,12,13,14]. The American Academy of Ophthalmology (AAO) recognizes the utility of ASEDs but emphasizes the need for further research to develop standardized protocols and long-term efficacy [12]. In addition, preparation methods and ASED concentrations have varied widely, hence standardization of production remains an ongoing challenge [1,2,15,16].

Our clinic produces ASEDs as preservative-free, reclosable, single-day use ophtioles on a per-patient basis, with dilution schemes (undiluted or 50% diluted with balanced salt solution) adapted to the patient’s indication and therapeutic response [17]. However, preservative-free eye drops carry a risk of contamination during use, most commonly involving bacteria and fungi in rarer cases [18]. The most frequently isolated organisms are coagulase-negative staphylococci (e.g., *Staphylococcus epidermidis*), and occasionally, other skin flora and fungal species, such as *Aspergillus* spp., are isolated as well. Contamination rates are highly dependent on the proper storage, handling, and duration of use [19,20]. Conventional containers without filters can have contamination rates of up to 29% after seven days, while containers with sterilizing filters or a one-way valve systems significantly reduce the risk, often to below 2% over several weeks of use [20,21].

The European Pharmacopoeia (Ph. Eur.) requires that multidose ophthalmic preparations incorporate antimicrobial agents or preservatives, or alternatively, be equipped with filtration systems or non-return valves to mitigate the risk of microbial contamination once opened. In contrast, single-day vials are not designed with such protective measures.

Ph. Eur. also states that multidose containers for eye drops must either contain antimicrobial agents or preservatives or alternatively be equipped with filters or valves in order to minimize the risk of microbial contamination after opening. Such protective systems are absent in vials intended for single-day use. These vials are nevertheless widely used as conventional lubricating eye drops, as they lower the likelihood of adverse reactions associated with preservatives. With few exceptions, however, their use is approved exclusively for single-use applications.

Single-day use with proper storage (refrigeration at 2–8 °C) is associated with very low contamination rates, typically under 2% for bacteria and fungi, and clinical infection is exceedingly rare, even when contamination is detected [22,23]. Longer-term use or improper handling increases risk, particularly if bottles are reused or exposed to non-sterile environments [20,24].

This study aimed to analyze the behavior of specific bacteria and fungi in the case of contamination during the of single-day use ophtioles containing unpreserved ASED stored at 2–8 °C.

## 2. Materials and Methods

This is an investigator-initiated, prospective, experimental study conducted at the Department of Ophthalmology at the USZ, Switzerland, and a regulatory-licensed, independent, external laboratory. The laboratory is licensed by Swissmedic for testing the sterility of medicinal products (H/V 1.6.1) and medical devices and is International Organization for Standardization (ISO) 17025 (International Organization for Standardization, Geneva, Switzerland, 2017) accredited, Good Manufacturing Practices (GMP) certified, and Food and Drug Administration (FDA) registered.

**Figure 1:**
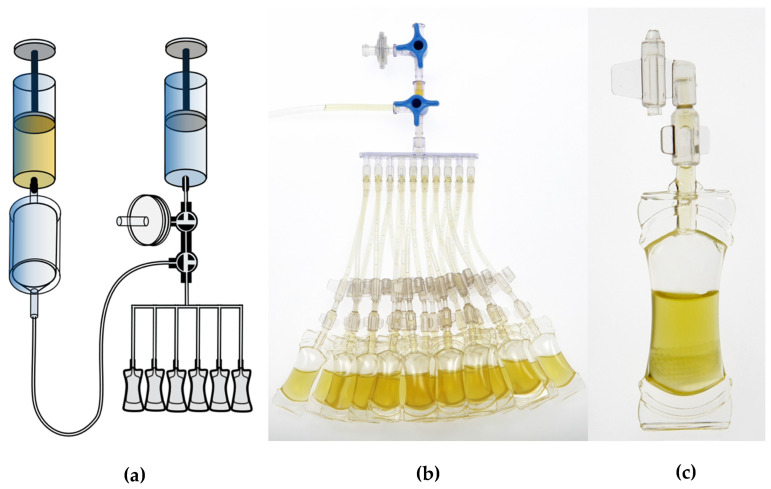
(**a**) Process of filling the single-day use ophtioles with unpreserved ASED using a prefiltered closed system. (**b**) Showing the filled ophtioles from a single manufacturing batch. (**c**) Image of a single-day use ophtiole in use, plugged with the cap.

**Figure 2:**
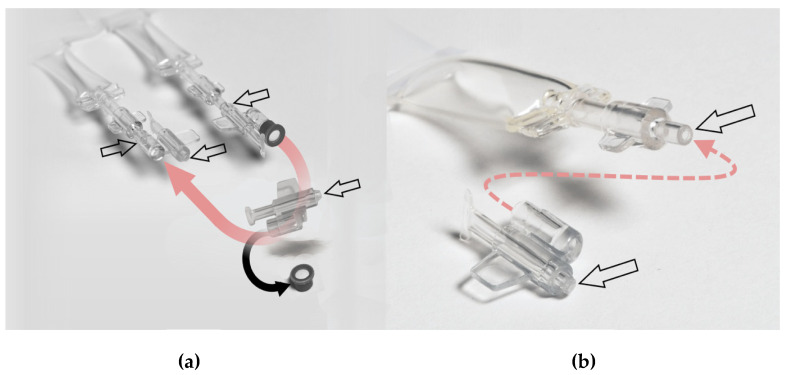
(**a**) The image displays an unopened ophtiole on the right. The arrow denotes the pre-determined breaking point, which, upon being reached, will result in the separation of the container from the closure cap. The opening of the closure cap is equipped with a sterile filter (black), which is discarded after opening the ophtiole. As illustrated on the left, the ophtiole is opened and then closed reversibly with the cap following the removal of the filter (see below). The arrows denote the pre-determined breaking points prior to and following the opening process. (**b**) The illustration provides a comprehensive depiction of an open ophtiole and its corresponding closure cap. Its sterile filter, not depicted, was previously removed. The arrows demarcate the predetermined breaking points following the opening process.

The study investigated single-day use ophtioles that contained ASEDs, which were produced based on the current guidelines of the Ph. Eur. and in compliance with the current GMP regulations. The sterility assessment method used was membrane filtration, which was also employed in the daily production routine. The filling process of the single-day use ophtioles with unpreserved ASEDs using a prefiltered closed system is illustrated in Figure 1, while the detailed structure of the ophtiole is demonstrated in Figure 2. Ethical approval was waived as this study did not fall within the scope of the Swiss Federal Human Research Act (BASEC-Nr. Req-2023-01239).

The ISO 17025-accredited, GMP-certified and FDA-registered laboratory performed microbial testing on the ASEDs in 100% concentration or 50% diluted from the original 2.5 mL ASED ophtioles, as provided by the Eye Bank of the Department of Ophthalmology, University Hospital of Zurich (USZ). The ophtioles are then inoculated with defined concentrations of 11 different bacterial and fungal strains, which were determined to be relevant in the case of possible contamination: *Staphylococcus (S.) aureus*, *Staphylococcus (S.) epidermidis*, *Streptococcus (S.) pyogenes*, *Bacillus (B.) subtilis*, *Micrococcus luteus*, *Cutibacterium (C.) acnes*, *Clostridium (C.) sporogenes*, *Escherichia (E.) coli*, *Pseudomonas (P.) aeruginosa*, *Candida (C.) albicans* and *Aspergillus (A.) brasiliensis* (see Supplements List S1). The specific test strains for *P. aeruginosa* and *B. subtilis* used in our experiment were *ATCC P. paraeruginosa* and *ATCC B. spizizenii*, respectively.

The test strains were chosen with regard to the following aspects, respecting the rules of the Ph. Eur.: the relevance as a contaminant (origin: hands, eye area, environment), its possibility of rapid growth under application or storage conditions (e.g., 20–25 °C, possibly 4 °C), and the coverage of various physiological groups (Gram-negative bacteria, Gram-positive bacteria, yeasts, molds) [25].

The inoculated samples were then stored at 2–8 °C or 20–25 °C. The microbial assessment was conducted at the following time points: 0, 3, 6, 9, 12, 24, and 48 h after inoculation, and at inoculation. As a positive control, ophtioles filled with nutrient medium instead of the product to be tested (e.g., ASED) are inoculated with the same microorganisms and stored at room temperature. The positive controls were tested at 0, 24 and 48 h.

### 2.1. Method Verification

Before conducting the microbial challenge test, the test method was verified for the specific product.

First, suspensions of 10^2^–10^3^ colony forming units (cfu)/mL in buffered sodium chloride-peptone solution (NPP) for each of the 11 test strains was prepared. Then, in order to quantify the inoculum, 2 × 0.1 mL of each tested strain suspension was spread separately on an agar plate (bacterial strains on Tryptone soya agar (TSA), except *C. sporogenes* on Columbia agar with 5% sheep blood (COS), and fungal strains on Sabouraud glucose agar (SGA)). Furthermore, for all test strains (except *C. sporogenes*), a pipet with 0.1 mL of the product dilution was applied twice on empty Petri dishes. Then 0.1 mL of the test strain suspension was added. Afterwards, 15–20 mL of liquefied TSA was added to the dishes with bacterial strains and 15–20 mL of liquefied SGA to the dishes with fungal strains. For *C. sporogenes*, a pipet with 0.1 mL of the product was applied twice onto COS. Then, 0.1 mL of the *C. sporogenes* suspension was added.

For negative product controls, the following plates were prepared: 2 × 0.1 mL product were pipetted onto an empty Petri dish, and 15–20 mL of liquified TSA was added, 2 × 0.1 mL product was pipetted onto an empty Petri dish and 15–20 mL of liquified SGA was added, and 2 × 0.1 mL product was spread on COS. As negative controls, the same plates were prepared, but instead of the product dilution, NPP was used.

All TSA and all COS plates were incubated at 30–35 °C for up to 3 days, and all SGA plates at 20–25 °C for up to 3 days. The TSA and SGA plates were incubated aerobically. The COS plates inoculated with *C. sporogenes* were incubated anaerobically. The counts for each of the 11 test organisms in the presence of the product are not allowed to differ by a factor greater than 2 from the respective inocula. In the case of microbial growth on the negative product control, the respective colonies need to be clearly distinguishable from the test strains. The negative controls must show no growth.

### 2.2. Microbial Challenge Test

Test strain suspensions of 10^7^–10^8^ cfu/mL in NPP of each of the 11 test strains were prepared. To quantify the inocula, the suspension was diluted to 10^2^–10^3^ cfu/mL and separately spread by 2 × 0.1 mL test strain suspension on TSA plates (respectively on COS for *C. sporogenes*).

#### 2.2.1. Product Samples Inoculated with Test Strains

**Figure 3:**
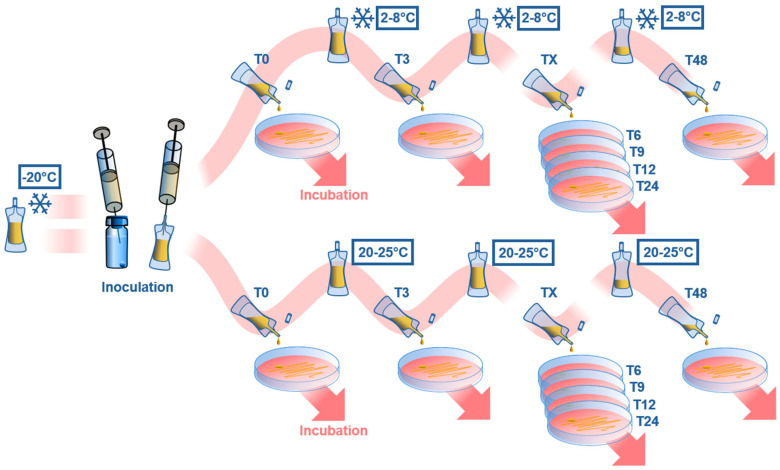
Simplified illustration providing an overview of the experimental workflow. First, autologous serum eye drop (ASED) ophtioles, containing 2.5 ml of 100% or 50% ASED are unfrozen. Then, these ophtioles were inoculated with 25 µL of the test strain, containing 10^5^–10^6^ colony-forming units (cfu). Afterwards, 0.1 ml of the inoculated ASED ophtiole was diluted with 10 mL buffered sodium chloride-peptone solution (NPP). Then 2 × 1 ml of this diluted, inoculated ASED sample was spread on a Petri dish at T0 for incubation. This was followed by storing the ASED ophtioles at 2–8 °C or 20–25 °C in order to repeat the same incubation process on Petri dishes at T3, T6, T9, T12, T24 and T48.

These samples contain ASEDs and were inoculated with test strains. Two ophtioles per test strain were inoculated with 25 μL of a strain suspension (inoculation of the product: 10^5^–10^6^ cfu/mL). Half of the inoculated ophtioles were kept at 2–8 °C and the other half at 20–25 °C (one test strain per temperature). Every test sample was analyzed at 0, 3, 6, 9, 12, 24, and 48 h. T0 is a standardized timepoint after inoculation. A total of 0.1 mL of the test sample was added to 10 mL of NPP to obtain a 1:100 dilution of the sample. Further dilutions were prepared and mixed by vortexing. Then, 2 × 1 mL of every sample dilution was separately pipetted into empty Petri dishes. Furthermore, 15–20 mL of liquefied TSA was added to the sample dilutions with bacterial strains, and liquefied SGA was added to the sample dilutions with fungal strains. For the sample with *C. sporogenes*, 2 × 0.1 mL of the sample was spread as a dilution on COS plates (Figure 3).

#### 2.2.2. Product Negative Controls

These samples contained ASEDs and were not inoculated. They were stored at 2–8 °C or 20–25 °C. The negative samples were analyzed at 0, 3, 6, 9, 12, 24, and 48 h. Then, 2 × 0.1 mL of the ophtiole was spread on COS plates, and 4 × 0.1 mL were separately pipetted from the ophtiole into empty Petri dishes. On two of the four plates, 15–20 mL of liquefied TSA was added; to the other two, liquefied SGA was added. All the TSA and all COS plates were incubated at 30–35 °C for maximal 5 days and all SGA plates at 20–25 °C for a maximum of 7 days. The TSA and SGA plates were incubated aerobically. The COS plates were incubated anaerobically. Then the cfu/mL was calculated.

#### 2.2.3. Samples with Nutrient Medium Inoculated with Test Strains (Positive Controls)

Samples with nutrient medium were inoculated with test strains. In the case of *C. sporogenes*, an ophtiole containing Thioglycollate (THIO) broth was inoculated with 25 μL of the test strain suspension of *C. sporogenes*. The inoculation of the nutrient medium contains 10^5^–10^6^ cfu/mL. Ten ophtioles were inoculated containing Casein–Peptone Soymeal-Peptone (CASO) broth with 25 μL of one of the remaining 10 test strain suspensions. The inoculated ophtioles were stored at 20–25 °C. Every sample was analyzed at 0, 24, and 48 h. Then, 0.1 mL of the test sample was added to 10 mL NPP to obtain a 1:100 dilution of the sample. Further dilutions were prepared and mixed by vortexing. A total of 2 × 1 mL of every sample dilution was separately pipetted into empty Petri dishes. Then, 15–20 mL of liquefied TSA was added to the sample dilutions with bacterial strains and liquefied SGA to the sample dilutions with fungal strains. For the sample with *C. sporogenes*, 2 × 0.1 mL of the sample were spread as a dilution on COS plates.

#### 2.2.4. Samples with Nutrient Medium (Negative Controls)

These samples did not contain ASEDs, were filled with nutrient medium, and were not inoculated. One ophtiole contained THIO broth and one ophtiole containing CASO broth. They were stored at 20–25 °C. The samples were analyzed at 0, 24, and 48 h. Then, 2 × 0.1 mL of the ophtiole containing THIO broth were separately spread on COS plates, and 4 × 0.1 mL of the ophtiole containing CASO broth were separately pipetted into empty Petri dishes. Two of the four plates were added 15–20 mL of liquefied TSA, and to the other two liquefied SGA.

#### 2.2.5. Evaluation

When all tests were performed, the initial microbial count of the product samples and the positive controls was calculated using the determined inocula. The initial microbial count was then compared with the microbial count measured at the different time points for every test strain by calculating the log level difference. The microbial count could increase, reduce, or not change. Product negative controls were expected to show no microbial growth.

#### 2.2.6. Acceptance Criteria

The count of the microorganism in the product should not increase over 24 h or 48 h, respectively. This means that the log level reduction should be ≥0.

#### 2.2.7. Risk Assessment

ASEDs, even though tested for several pathogens, must be considered potentially infectious. Therefore, when processing the product, the technicians adhered to the following safety precautions: avoiding skin contact—hence, gloves were worn at all times—and the product dilutions were autoclaved before disposal. With these precautions in place, the analysis was performed without increased risk for the technicians.

## 3. Results

The tests were performed from August to October 2025 at the independent laboratory upon receipt of the samples from the USZ.

The acceptance criteria for this study are that the count of the microorganism in the product should not increase over 24 h and 48 h when stored at 2–8 °C (log value reduction was ≥0).

**Figure 4:**
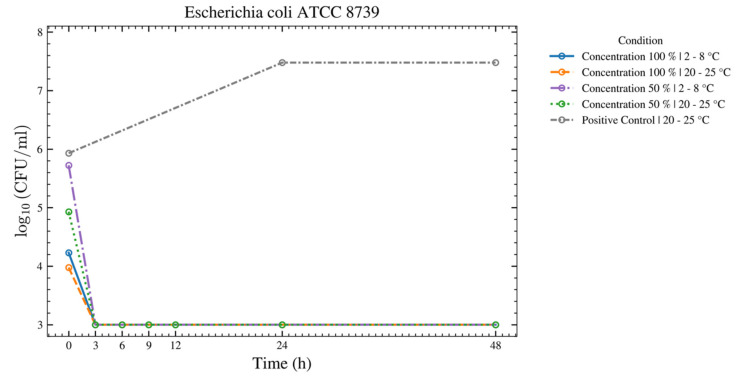
This figure provides a results overview of the tests performed with the test strain of *E. coli* using autologous serum eye drops (ASEDs) that contained 100% and 50% concentrations and were stored at 2–8 °C and 20–25 °C. The positive control that contained only the nutrient medium is shown as well. The upper threshold value was defined as log_10_ 7.5; therefore, values of 3 × 10^7^ colony-forming units (CFU) cannot be distinguished from higher values in this figure. Similarly, a value of log_10_ 3.0 was defined as the lower threshold, meaning values of 1 × 10^3^ CFU are indistinguishable from lower values in this figure.

**Figure 5:**
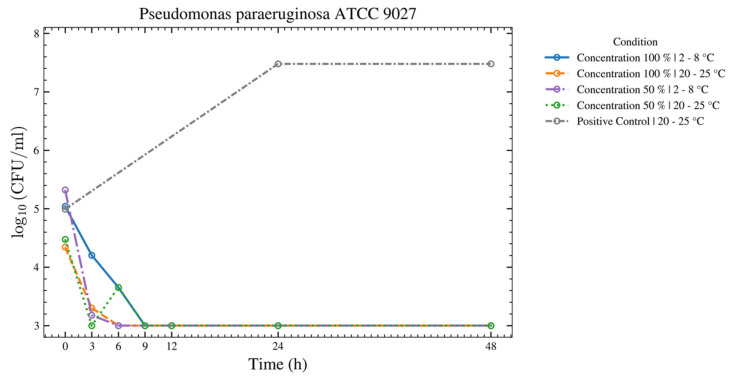
This figure provides a results overview of the tests performed with the test strain of *P. paraeruginosa* using autologous serum eye drops (ASEDs) that contained 100% and 50% concentrations and were stored at 2–8 °C and 20–25 °C. The positive control that contained only the nutrient medium is shown as well. The upper threshold value was defined as log_10_ 7.5; therefore, values of 30,000,000 colony-forming units (CFU) cannot be distinguished from higher values in this figure. Similarly, a value of log_10_ 3.0 was defined as the lower threshold, meaning values of 1 × 10^3^ CFU are indistinguishable from lower values in this figure.

Seven species—*C. sporogenes*, *B. spizizenii*, *M. luteus*, *A. brasiliensis*, *C. acnes*, *P. aeruginosa*, and *E. coli*—showed no increase over 48 h at both temperatures for the 50% and 100% ASEDs (Figure 4 and Figure 5). Four of these seven strains—*C. sporogenes*, *B. spizizenii*, *P. aeruginosa*, and *E. coli*—exhibited a strong log reduction within the first 12 h at 2–8 °C, followed by an absence of growth until 48 h.

**Figure 6:**
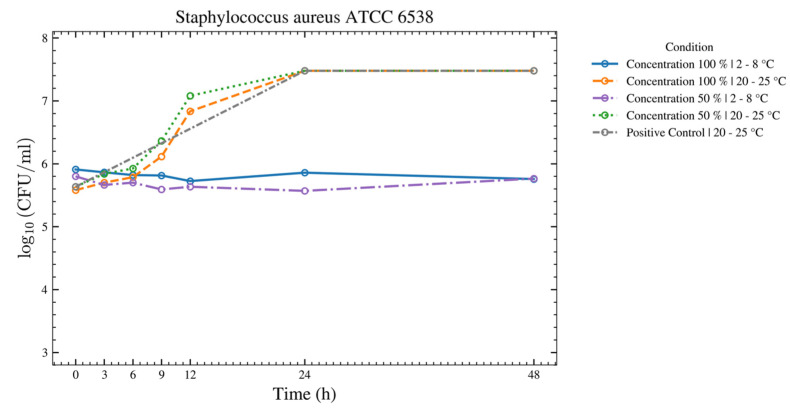
This figure provides a results overview of the tests performed with the test strain of *S. aureus* using autologous serum eye drops (ASEDs) that contained 100% and 50% concentrations and were stored at 2–8 °C and 20–25 °C. The positive control that contained only the nutrient medium is shown as well. The upper threshold value was defined as log_10_ 7.5; therefore, values of 3 × 10^7^ colony-forming units (CFU) cannot be distinguished from higher values in this figure. Similarly, a value of log_10_ 3.0 was defined as the lower threshold, meaning values of 1 × 10^3^ CFU are indistinguishable from lower values in this figure.

**Figure 7:**
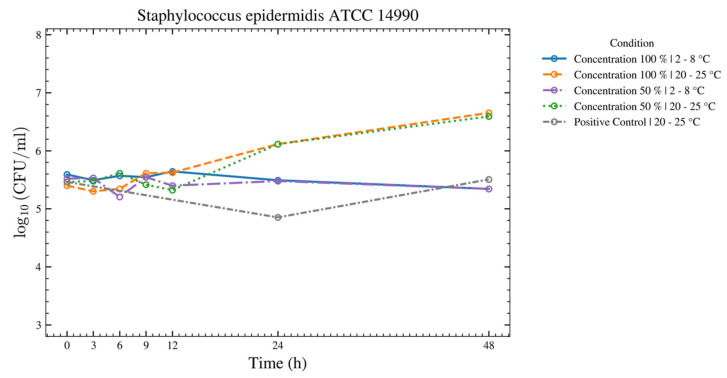
This figure provides an overview of the tests performed with the test strain of *S. epidermidis* using autologous serum eye drops (ASEDs) that contained 100% and 50% concentrations and were stored at 2–8 °C and 20–25 °C. The positive control that contained only the nutrient medium is shown as well.

**Figure 8:**
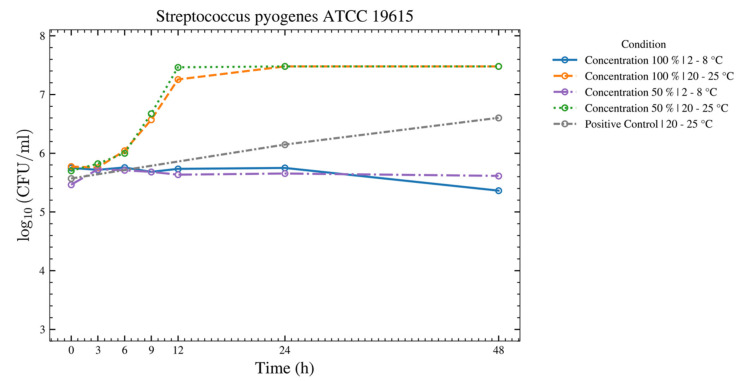
This figure provides an overview of the tests performed with the test strain of *S. pyogenes* using autologous serum eye drops (ASEDs) that contained 100% and 50% concentrations and were stored at 2–8 °C and 20–25 °C. The positive control that contained only the nutrient medium is shown as well. The upper threshold value was defined as log_10_ 7.5; therefore, values of 3 × 10^7^ colony-forming units (CFU) cannot be distinguished from higher values in this figure.

**Figure 9:**
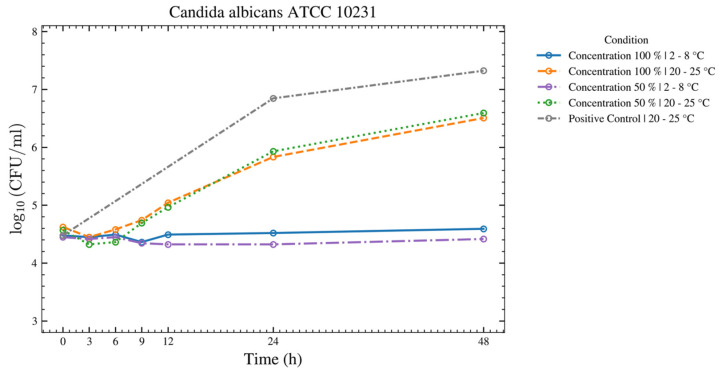
This figure provides an overview of the tests performed with the test strain of *C. albicans* using autologous serum eye drops (ASEDs) that contained 100% and 50% concentrations and were stored at 2–8 °C and 20–25 °C. The positive control that contained only the nutrient medium is shown as well.

For *S. aureus*, *S. epidermidis*, *S. pyogenes*, and *C. albicans* (Figure 6, Figure 7, Figure 8 and Figure 9), there was no increase over 48 h at 2–8 °C for the 50% and 100% ASEDs. At room temperature, both concentrations showed microbial growth.

The negative controls showed no growth as expected, whereas the positive controls showed no inhibition.

## 4. Discussion

This prospective study evaluated the microbial growth of 11 test strains, including bacterial and fungal species that were identified as potential contaminants following the opening of single-day use ophtioles that contained 50% and 100% ASEDs. All 11 microorganisms met the predefined acceptance criteria between T0 and T24, as well as T48, when stored at 2–8 °C. For seven microorganisms, the acceptance criteria were fulfilled at both 2–8 °C and 20–25 °C over the same period. In contrast, four microorganisms—*C. sporogenes*, *B. spizizenii*, *P. aeruginosa*, and *E. coli*—met the criteria only under refrigerated conditions (2–8 °C) from T0 to T24 and up to T48. As anticipated, the negative controls exhibited no growth, while the positive controls demonstrated either no inhibition or even growth. These findings support the microbiological safety of reclosable single-day use ophtioles containing preservative-free ASEDs after opening when stored at 2–8 °C.

The main safety concern with ASEDs is the potential for microbial contamination during preparation, storage, or use. Cases of microbial growth in containers and rare instances of conjunctivitis have been reported, underscoring the need for a strict aseptic technique during preparation and handling [2,3]. The contamination risk associated with different ASED concentrations has not been analyzed in greater detail, although it does not seem to differ with concentration [22,23,26]. The microbial safety of manufactured ASEDs can be ensured until the container is opened by means of aseptic manufacturing and sterility testing. However, container contamination after opening poses a potential risk, especially for reclosable single-day use ophtioles. To our knowledge, this is the first study evaluating the microbial behavior of different possible contamination strains in single-day use ophtioles and ASEDs.

The AAO specifically highlights the importance of sterility in preparation protocols to minimize infection risk, as ASEDs themselves apparently do not seem to prevent microbial growth [10]. The composition of ASEDs is similar to natural tears, containing growth factors, vitamins, and proteins. However, the antimicrobial activity of ASEDs is controversial. Current literature notes that unlike natural tears, ASEDs are not bacteriostatic and do not contain lysozyme or lactoferrin at physiologically relevant concentrations for antimicrobial defense [2,4].

In our study, the microbial concentration decreased over time in some of the test strains, suggesting antimicrobial activity in ASEDs. Serum is a complex biological solution containing several components that can contribute to bacterial killing, mainly the complement system, natural antibodies, including IgG and IgM, and antimicrobial enzymes, such as lysozyme. The complement system is composed of several plasma proteins that become activated when they encounter pathogens or pathogen-bound antibodies, triggering a cascade of reactions that leads to opsonization, inflammation, and bacterial lysis. This mechanism is particularly effective against Gram-negative bacteria. Lysozyme, a small antimicrobial enzyme found in body fluids such as tears, acts as a natural antibiotic by degrading bacterial cell walls, especially in Gram-positive bacteria. Thus, the effect of the serum on the bacterial growth is multifactorial and often temperature-dependent. It is possible that specific antibodies against some of these microorganisms are present in the serum [27]. Complement was first discovered in the 1980s when it was found to aid or “complement” the killing of bacteria by heat-stable antibodies present in normal serum [28]. Given that bacteria are killed by complement proteins within the blood (or serum), this process is referred to as serum killing [29]. Thus, the serum killing effect, or serum bactericidal activity (SBA), is the ability of complement proteins in the serum to kill bacteria, particularly Gram-negative ones, by forming a membrane attack complex (MAC) that ruptures the pathogen’s membrane. It acts as a crucial, innate immune defense, typically measured by testing the serum’s ability to destroy > 99.9% of a bacterial inoculum. During the preparation of serum eye drops, the serum is sterile-filtered using a 0.22 µm (220 nm) PES filter. The largest serum proteins are approximately 12–16 nm in size, thereby, serum proteins easily pass through the filter, remaining in the final product.

As shown in Figure 4, after 3 h, a strong log reduction in *E. coli* at both temperatures and both concentrations of serum was observed. Between 3 h and 48 h, no growth of *E. coli* was observed. A serum killing effect on *E. coli* has been well-described in the literature [29]. Despite the ability of *P. paraeruginosa* to grow at 4 °C, our experiment showed a strong log reduction within the first 9 h, and later on, the absence of growth until 48 h, thus suggesting that the serum killing effect might still hinder the bacterial growth over time (Figure 5). For *C. albicans*, growth was observed at 20–25 °C, while none was observed at 2–8 °C. As *C. albicans* does not actively grow at 4 °C (its optimal growth temperature being 33–37 °C), the absence of growth in both serum concentrations at 2–8 °C may be solely due to the cooling effect, rather than a true serum killing effect (Figure 9). Nevertheless, this finding highlights the ASED safety regarding *C. albicans*, if stored at the low temperature of 4 °C. In summary, ASEDs might have a possible bactericidal effect. Further investigations are needed to elaborate this matter in greater detail.

Regarding bacterial overgrowth—*S. aureus*, *S. epidermidis*, and *S. pyogenes*—have a thick Gram-positive cell wall, as does the fungus *C. albicans*. A major defense mechanism of human serum is the membrane attack complex (MAC), which is formed after deposition of complement proteins on the bacterial surface [30]. This so-called serum bactericidal effect typically targets Gram-negative bacteria, in which the MAC disrupts the outer membrane, a structure that is absent in Gram-positive bacteria. In addition, the pathogenic species showing overgrowth are well adapted to survive in the human host environment, unlike commensal species, such as *B. subtilis* and *M. luteus*, which are also Gram-positive but generally exist in a balanced relationship with the host and are therefore more effectively controlled by host defense mechanisms.

This study has several limitations. First, all the experiments were conducted at a single study site, which may limit the generalizability of the findings. Laboratory conditions, handling procedures, environmental factors, and local standard operating procedures may differ across institutions. Regarding the microbial count, it should not be overlooked that determining the microbial count is not an exact method in itself but is subject to variability. Moreover, certain strains, e.g., *C. acnes*, only grow slowly aerobically, further limiting the method of microbial counting. For the positive control, no analyses were carried out at time points of 3, 6, 9, and 12 h, so the growth is shown as linear between 0 h and 24 h. However, this can hide the real growth variations during this interval.

## 5. Conclusions

In this prospective microbial challenge study, reclosable single-day use ophtioles containing preservative-free ASEDs showed no relevant microbial growth at 2–8 °C across 11 bacterial and fungal strains in both 50% and 100% concentrations from T0 to T24 and remained stable up to T48 under refrigeration. Four of these strains exhibited a strong log reduction within the first 12 h at 2–8 °C, followed by an absence of growth until 48 h. In contrast, several organisms demonstrated growth at 20–25 °C, highlighting temperature as the key determinant of microbiological growth. These findings support the microbiological safety of reclosable single-day use ophtioles containing preservative-free ASED after opening when stored at 2–8 °C and emphasize strict refrigerated storage during use.

## Data Availability

The original contributions presented in this study are included in the article/Supplementary Materials. Further inquiries can be directed to the corresponding author.

## Supplementary Materials

The following supporting information can be downloaded at https://www.mdpi.com/article/10.3390/bioengineering13040417/s1, List S1: Microbial strains, Solutions and media, Devices, instruments and other equipment; Table S1: Microbial enumeration of *Streptococcus pyogenes* ATCC 19615.; Table S2: Microbial enumeration of *Clostridium sporogenes* ATCC 19404.; Table S3: Microbial enumeration of *Bacillus spizizenii* ATCC 6633.; Table S4: Microbial enumeration of *Micrococcus luteus* ATCC 10240; Table S5: Microbial enumeration of *Staphylococcus epidermidis* ATCC 14990; Table S6: Microbial enumeration of *Candida albicans* ATCC 10231; Table S7: Microbial enumeration of *Aspergillus brasiliensis* ATCC 16404; Table S8: Microbial enumeration of *Cutibacterium acnes* ATCC 8919; Table S9: Microbial enumeration of *Pseudomonas paraeruginosa* ATCC 9027; Table S10: Microbial enumeration of *Escherichia coli* ATCC 8739; Table S11: Microbial enumeration of *Staphylococcus aureus* ATCC 6538.

## Abbreviations

The following abbreviations are used in this manuscript:

AAO	American Academy of Ophthalmology
ASED	Autologous serum eye drops
NPP	Buffered sodium chloride-peptone solution pH 7.0
CASO	Casein soya bean digest (CASO)
COS	Columbia agar with 5% sheep blood plates
SGA	Sabouraud glucose agar
NaCl	Sodium chloride
THIO	Thioglycollate
TSA	Tryptone soya agar
