# Supplementary material for: Microbial Assessment of Reclosable Single-Day Use Ophtioles Containing Autologous Serum Eye Drops
Source: Bioengineering (Basel). 2026 Apr 2;13(4):417. doi: 10.3390/bioengineering13040417 (PMC13114155; doi:10.3390/bioengineering13040417)
Supplement: Supplementary file 1 [file bioengineering-13-00417-s001.zip › bioengineering-4173645-supplementary.pdf]

bioengineering

Supplementary Materials

List S1:

Microbial strains

1. Gram-positive bacteria

Staphylococcus aureus ATCC 6538

Staphylococcus epidermidis ATCC 14990

Streptococcus pyogenes ATCC 19615

Bacillus subtilis ATCC 6633 (Test strain is *Bacillus spizizenii*)

Micrococcus luteus ATCC 10240

Cutibacterium acnes ATCC 6919

Clostridium sporogenes ATCC 19404

2. Gram-negative bacteria

Pseudomonas aeruginosa ATCC 9027 (Test strain is *Pseudomonas paraeruginosa*)

Escherichia coli ATCC 8739

3. Yeast

Candida albicans ATCC 10231

4. Mold

Aspergillus brasiliensis ATCC 16404

Solutions and media

The following Solutions and media were used:

- Buffered sodium chloride-peptone solution pH 7.0 (NPP)

- Casein soya bean digest (CASO) broth

- Columbia agar with 5% sheep blood (COS) plates

- Sabouraud glucose agar (SGA) liquefied (max. 45 °C)

- Sabouraud glucose agar (SGA) plates

- Sodium chloride solution 0.86 % (NaCl)

- Thioglycollate (THIO) broth

- Tryptone soya agar (TSA) liquefied (max. 45 °C)

- Tryptone soya agar (TSA) plates

Devices, instruments and other equipment

The following devices were used:

- Densitometer (DensiCHEK Plus, bioMérieux)

- Refrigerator (2-8 °C); temperature monitored

- Incubator (20-25 °C); temperature monitored

- Petri dishes (diameter 9 cm)

- Standard laboratory equipment

https://doi.org/10.3390/bioengineering13040417

Table S1: Microbial enumeration of *Streptococcus pyogenes* ATCC 19615.

***Streptococcus pyogenes* ATCC 19615**	**Temperature (°C)**	**Inoculum (cfu/mL)**	**0h (cfu/mL)**	**3h (cfu/mL)**	**6h (cfu/mL)**	**9h (cfu/mL)**	**12h (cfu/mL)**	**24h (cfu/mL)**	**z**	**48 h (cfu/mL)**
Concentration 100 %	2 - 8	4.0E+05	5.6E+05	5.2E+05	5.7E+05	4.8E+05	5.4E+05	5.6E+05		2.3E+05
Log reduction to inoculum			0	0	0	0	0	0		0
Log reduction to 0h				0	0	0	0	0		0
Concentration 100 %	20 - 25	4.0E+05	5.9E+05	5.7E+05	1.1E+06	3.7E+06	1.8E+07	>3E+07		> 3E+07
Log reduction to inoculum			0	0	0	-1	-2	<-2		<-2
Log reduction to 0h				0	0	-1	-1	<-2		<-2
Concentration 50 %	2 - 8	4.0E+05	2.9E+05	5.3E+05	5.1E+05	4.8E+05	4.3E+05	4.5E+05		4.1E+05
Log reduction to inoculum			0	0	0	0	0	0		0
Log reduction to 0h				0	0	0	0	0		0
Concentration 50 %	20 - 25	4.0E+05	5.0E+05	6.6E+05	1.0E+06	4.7E+06	2.9E+07	> 3E+07		> 3E+07
Log reduction to inoculum			0	0	0	-1	-2	<-2		<-2
Log reduction to 0h				0	0	-1	-2	<-2		<-2
Positive Control	20 - 25	4.0E+05	3.7E+05	n.a	n.a	n.a	n.a	1.4E+06		4.0E+06

Abbreviations: Colony-forming unit (cfu).

https://doi.org/10.3390/bioengineering13040417

Table S2: Microbial enumeration of *Clostridium sporogenes* ATCC 19404.

***Clostridium sporogenes* ATCC 19404**	**Temperature (°C)**	**Inoculum (cfu/mL)**	**0h (cfu/mL)**	**3h (cfu/mL)**	**6h (cfu/mL)**	**9h (cfu/mL)**	**12h (cfu/mL)**	**24h (cfu/mL)**	**48 h (cfu/mL)**
Concentration 100 %	2 - 8	4.1E+05	4.6E+04	1.0E+03	1.0E+03	<1000	1.0E+03	1.0E+03	1.0E+03
Log reduction to inoculum			1	3	3	>3	3	3	3
Log reduction to 0h				2	2	>2	2	2	2
Concentration 100 %	20 - 25	4.1E+05	3.5E+04	<1000	<1000	<1000	<1000	<1000	<1000
Log reduction to inoculum			1	1	>3	>3	>3	>3	>3
Log reduction to 0h				>2	>2	>2	>2	>2	>2
Concentration 50 %	2 - 8	4.1E+05	3.9E+04	3.0E+03	2.0E+03	<1000	1.0E+03	<1000	<1000
Log reduction to inoculum			1	2	2	>3	3	>3	>3
Log reduction to 0h				1	1	>2	2	>2	>2
Concentration 50 %	20 - 25	4.1E+05	5.5E+04	<1000	1.0E+03	<1000	<1000	<1000	<1000
Log reduction to inoculum			1	>3	3	>3	>3	>3	>3
Log reduction to 0h				>2	2	>2	>2	>2	>2
Positive Control	20 - 25	4.1E+05	1.4E+05	n.a	n.a	n.a	n.a	2.6E+04	1.1E+04

Abbreviations: Colony-forming unit (cfu).

Table S3: Microbial enumeration of *Bacillus spizizenii* ATCC 6633.

***Bacillus spizizenii* ATCC 6633**	**Temper-ature (°C)**	**Inoculum (cfu/mL)**	**0h (cfu/mL)**	**3h (cfu/mL)**	**6h (cfu/mL)**	**9h (cfu/mL)**	**12h (cfu/mL)**	**24h (cfu/mL)**	**48 h (cfu/mL)**
Concentration 100 %	2 - 8	3.2E+05	1.2E+05	1.6E+04	2.0E+03	3.0E+03	4.5E+03	2.0E+03	1.0E+03
Log reduction to inoculum			0	1	2	2	2	2	3
Log reduction to 0h				1	2	2	1	2	2
Concentration 100 %	20 - 25	3.2E+05	1.0E+00	1.0E+00	1.7E+04	1.6E+04	1.0E+03	<1000	<1000
Log reduction to inoculum			1	1	1	1	3	>3	>3
Log reduction to 0h				0	1	1	2	>2	>2
Concentration 50 %	2 - 8	3.2E+05	1.0E+05	1.5E+04	9.0E+03	4.0E+03	5.0E+03	3.0E+03	4.0E+03
Log reduction to inoculum			1	1	2	2	2	2	2
Log reduction to 0h				1	1	1	1	2	1
Concentration 50 %	20 - 25	3.2E+05	9.2E+04	3.4E+04	2.0E+04	7.0E+03	<1000	<1000	6.0E+03
Log reduction to inoculum			1	1	1	2	>3	>3	2
Log reduction to 0h				0	1	1	>2	>2	1
Positive Control	20 - 25	3.2E+05	5.1E+05	n.a	n.a	n.a	n.a	1.7E+07	1.7E+08

Abbreviations: Colony-forming unit (cfu).

Table S4: Microbial enumeration of *Micrococcus luteus* ATCC 10240

***Micrococcus luteus* ATCC 10240**	**Temperature (°C)**	**Inoculum (cfu/mL)**	**0h (cfu/mL)**	**3h (cfu/mL)**	**6h (cfu/mL)**	**9h (cfu/mL)**	**12h (cfu/mL)**	**24h (cfu/mL)**	**48 h (cfu/mL)**
Concentration 100 %	2 - 8	3.8E+05	3.5E+05	3.4E+05	3.3E+05	4.9E+05	3.7E+04	3.2E+05	2.4E+05
Log reduction to inoculum			0	0	0	0	1	0	0
Log reduction to 0h				0	0	0	1	0	0
Concentration 100 %	20 - 25	3.8E+05	3.6E+05	2.9E+05	3.6E+05	3.4E+05	4.0E+05	3.8E+05	3.1E+05
Log reduction to inoculum			0	0	0	0	0	0	0
Log reduction to 0h				0	0	0	0	0	0
Concentration 50 %	2 - 8	3.8E+05	3.0E+05	2.7E+05	2.0E+05	3.7E+05	4.1E+05	3.1E+05	2.1E+05
Log reduction to inoculum			0	0	0	0	0	0	0
Log reduction to 0h				0	0	0	0	0	0
Concentration 50 %	20 - 25	3.8E+05	3.5E+05	2.5E+05	3.2E+05	2.3E+05	3.9E+05	3.3E+05	3.0E+05
Log reduction to inoculum			0	0	0	0	0	0	0
Log reduction to 0h				0	0	0	0	0	0
Positive Control	20 - 25	3.8E+05	3.9E+05	n.a	n.a	n.a	n.a	1.1E+07	1.0E+08

Abbreviations: Colony-forming unit (cfu).

https://doi.org/10.3390/bioengineering13040417

Table S5: Microbial enumeration of *Staphylococcus epidermidis* ATCC 14990.

***Staphylococcus epidermidis* ATCC 14990**	**Temperature (°C)**	**Inoculum (cfu/mL)**	**0h (cfu/mL)**	**3h (cfu/mL)**	**6h (cfu/mL)**	**9h (cfu/mL)**	**12h (cfu/mL)**	**24h (cfu/mL)**	**48 h (cfu/mL)**
Concentration 100 %	2 - 8	3.2E+05	3.9E+05	3.1E+05	3.7E+05	3.5E+05	4.4E+05	3.1E+05	2.2E+05
Log reduction to inoculum			0	0	0	0	0	0	0
Log reduction to 0h				0	0	0	0	0	0
Concentration 100 %	20 - 25	3.2E+05	2.5E+05	2.0E+05	2.2E+05	4.1E+05	4.2E+05	1.3E+06	4.5E+06
Log reduction to inoculum			0	0	0	0	0	-1	-1
Log reduction to 0h				0	0	0	0	-1	-1
Concentration 50 %	2 - 8	3.2E+05	3.3E+05	3.4E+05	1.6E+05	3.5E+05	2.5E+05	3.0E+05	2.2E+05
Log reduction to inoculum			0	0	0	0	0	0	0
Log reduction to 0h				0	0	0	0	0	0
Concentration 50 %	20 - 25	3.2E+05	2.9E+05	3.0E+05	4.1E+05	2.6E+05	2.1E+05	1.3E+06	3.9E+06
Log reduction to inoculum			0	0	0	0	0	-1	-1
Log reduction to 0h				0	0	0	0	-1	-1
Positive Control	20 - 25	3.2E+05	2.9E+05	n.a	n.a	n.a	n.a	7.1E+04	3.2E+05

Abbreviations: Colony-forming unit (cfu).

https://doi.org/10.3390/bioengineering13040417

Table S6: Microbial enumeration of *Candida albicans* ATCC 10231

***Candida albicans* ATCC 10231**	**Tempera-ture (°C)**	**Inoculum (cfu/mL)**	**0h (cfu/mL)**	**3h (cfu/mL)**	**6h (cfu/mL)**	**9h (cfu/mL)**	**12h (cfu/mL)**	**24h (cfu/mL)**	**48 h (cfu/mL)**
Concentration 100 %	2 -8	2.0E+05	3.0E+04	2.8E+04	3.1E+04	2.3E+04	3.1E+04	3.3E+04	3.9E+04
Log reduction to inoculum			1	1	1	1	1	1	1
Log reduction to 0h				0	0	0	0	0	0
Concentration 100 %	20 - 25	2.0E+05	4.2E+04	2.8E+04	3.8E+04	5.5E+04	1.1E+05	6.8E+05	3.2E+06
Log reduction to inoculum			1	1	1	1	0	-1	-1
Log reduction to 0h				0	0	0	0	-1	-2
Concentration 50 %	2 - 8	2.0E+05	2.8E+04	2.6E+04	2.8E+04	2.2E+04	2.1E+04	2.1E+04	2.6E+04
Log reduction to inoculum			1	1	1	1	1	1	1
Log reduction to 0h				0	0	0	0	0	0
Concentration 50 %	20 - 25	2.0E+05	3.7E+04	2.1E+04	2.3E+04	4.9E+04	9.2E+04	8.5E+05	3.9E+06
Log reduction to inoculum			1	1	1	1	0	-1	-1
Log reduction to 0h				0	0	0	0	-1	-2
Positive Control	20 - 25	2.0E+05	3.0E+04	n.a	n.a	n.a	n.a	7.0E+06	2.1E+07

Abbreviations: Colony-forming unit (cfu).

Table S7: Microbial enumeration of *Aspergillus brasiliensis* ATCC 16404

***Aspergillus brasiliensis* ATCC 16404**	**Temperature (°C)**	**Inoculum (cfu/mL)**	**0h (cfu/mL)**	**3h (cfu/mL)**	**6h (cfu/mL)**	**9h (cfu/mL)**	**12h (cfu/mL)**	**24h (cfu/mL)**	**48 h (cfu/mL)**
Concentration 100 %	2 - 8	2.3E+05	2.2E+04	1.3E+04	2.9E+04	1.9E+04	2.3E+04	1.9E+04	1.1E+04
Log reduction to inoculum			1	1	1	1	1	1	1
Log reduction to 0h				0	0	0	0	0	0
Concentration 100 %	20 - 25	2.3E+05	2.4E+04	2.9E+04	2.2E+04	2.4E+04	2.3E+04	2.4E+04	2.1E+04
Log reduction to inoculum			1	1	1	1	1	1	1
Log reduction to 0h				0	0	0	0	0	0
Concentration 50 %	2 - 8	2.3E+05	2.4E+04	2.3E+04	1.2E+04	1.9E+04	1.0E+04	1.3E+04	1.1E+04
Log reduction to inoculum			1	1	1	1	1	1	1
Log reduction to 0h				0	0	0	0	0	0
Concentration 50 %	20 - 25	2.3E+05	2.2E+04	2.8E+04	2.2E+04	1.8E+04	2.4E+04	1.2E+04	2.2E+04
Log reduction to inoculum			1	1	1	1	1	1	1
Log reduction to 0h				0	0	0	0	0	0
Positive Control	20 - 25	2.3E+05	2.2E+04	n.a	n.a	n.a	n.a	3.3E+04	2.6E+04

Abbreviations: Colony-forming unit (cfu).

https://doi.org/10.3390/bioengineering13040417

Table S8: Microbial enumeration of *Cutibacterium acnes* ATCC 8919

***Cutibacterium acnes* ATCC 8919**	**Temperature (°C)**	**Inoculum (cfu/mL)**	**0h (cfu/mL)**	**3h (cfu/mL)**	**6h (cfu/mL)**	**9h (cfu/mL)**	**12h (cfu/mL)**	**24h (cfu/mL)**	**48 h (cfu/mL)**
Concentration 100 %	2 - 8	3.1E+05	3.4E+05	4.1E+05	3.1E+04	3.7E+05	<1000	1.3E+04	3.2E+05
Log reduction to inoculum			0	0	1	0	>2	1	0
Log reduction to 0h				0	0	0	>3	1	0
Concentration 100 %	20 - 25	3.1E+05	3.7E+05	3.1E+05	3.2E+04	3.5E+05	<1000	9.9E+04	3.2E+05
Log reduction to inoculum			0	0	1	0	>2	0	0
Log reduction to 0h				0	1	0	>3	1	0
Concentration 50 %	2 - 8	3.1E+05	3.9E+05	3.1E+05	3.3E+05	3.1E+05	<1000	9.9E+04	1.5E+05
Log reduction to inoculum			0	0	0	0	>2	0	0
Log reduction to 0h				0	0	0	>3	1	0
Concentration 50 %	20 - 25	3.1E+05	4.0E+05	3.1E+05	3.2E+05	<1000	<1000	3.0E+05	3.4E+05
Log reduction to inoculum			0	0	0	>2	>2	0	0
Log reduction to 0h				0	0	>3	>3	0	0
Positive Control	20 - 25	3.1E+05	3.3E+04	n.a	n.a	n.a	n.a	3.3E+05	3.0E+05

Abbreviations: Colony-forming unit (cfu).

Table S9: Microbial enumeration of *Pseudomonas paraeruginosa* ATCC 9027

***Pseudomonas paraeruginosa* ATCC 9027**	**Temperature (°C)**	**Inoculum (cfu/mL)**	**0h (cfu/mL)**	**3h (cfu/mL)**	**6h (cfu/mL)**	**9h (cfu/mL)**	**12h (cfu/mL)**	**24h (cfu/mL)**	**48 h (cfu/mL)**
Concentration 100 %	2 - 8	5.9E+05	1.1E+05	1.6E+04	4.5E+03	1.0E+03	<1000	<1000	<1000
Log reduction to inoculum			0	2	2	3	>3	>3	>3
Log reduction to 0h				1	1	2	>2	>2	>2
Concentration 100 %	20 - 25	5.9E+05	2.2E+04	2.0E+03	<1000	<1000	<1000	<1000	<1000
Log reduction to inoculum			1	2	>3	>3	>3	>3	>3
Log reduction to 0h				1	>1	>1	>1	>1	>1
Concentration 50 %	2 - 8	5.9E+05	2.1E+05	1.5E+03	1.0E+03	<1000	<1000	<1000	<1000
Log reduction to inoculum			0	3	3	>3	>3	>3	>3
Log reduction to 0h				2	2	>2	>2	>2	>2
Concentration 50 %	20 - 25	5.9E+05	3.0E+04	<1000	4.5E+03	<1000	<1000	<1000	<1000
Log reduction to inoculum			1	>3	2	>3	>3	>3	>3
Log reduction to 0h				>1	1	>1	>1	>1	>1
Positive Control	20 - 25	5.9E+05	9.8E+04	n.a	n.a	n.a	n.a	>30000000	>30000000

Abbreviations: Colony-forming unit (cfu).

https://doi.org/10.3390/bioengineering13040417

Table S10: Microbial enumeration of *Escherichia coli* ATCC 8739

***Escherichia coli* ATCC 8739**	**Temperature (°C)**	**Inoculum (cfu/mL)**	**0h (cfu/mL)**	**3h (cfu/mL)**	**6h (cfu/mL)**	**9h (cfu/mL)**	**12h (cfu/mL)**	**24h (cfu/mL)**	**48 h (cfu/mL)**
Concentration 100 %	2 - 8	5.0E+05	1.7E+04	1.0E+03	<1000	<1000	<1000	<1000	<1000
Log reduction to inoculum			1	3	>3	>3	>3	>3	>3
Log reduction to 0h				1	>1	>1	>1	>1	>1
Concentration 100 %	20 - 25	5.0E+05	9.5E+03	<1000	<1000	<1000	<1000	<1000	<1000
Log reduction to inoculum			2	>3	>3	>3	>3	>3	>3
Log reduction to 0h				>1	>1	>1	>1	>1	>1
Concentration 50 %	2 - 8	5.0E+05	5.3E+05	1000	<1000	<1000	<1000	<1000	<1000
Log reduction to inoculum			0	>3	>3	>3	>3	>3	>3
Log reduction to 0h				>3	>3	>3	>3	>3	>3
Concentration 50 %	20 - 25	5.0E+05	8.5E+04	<1000	<1000	<1000	<1000	<1000	<1000
Log reduction to inoculum			1	>3	>3	>3	>3	>3	>3
Log reduction to 0h				>2	>2	>2	>2	>2	>2
Positive Control	20 - 25	5.0E+05	8.5E+05	n.a	n.a	n.a	n.a	>30000000	>30000000

Abbreviations: Colony-forming unit (cfu).

Table S11: Microbial enumeration of *Staphylococcus aureus* ATCC 6538

***Staphylococcus aureus* ATCC 6538**	**Temperature (°C)**	**Inoculum (cfu/mL)**	**0h (cfu/mL)**	**3h (cfu/mL)**	**6h (cfu/mL)**	**9h (cfu/mL)**	**12h (cfu/mL)**	**24h (cfu/mL)**	**48 h (cfu/mL)**
Concentration 100 %	2 - 8	2.2E+05	8.1E+05	7.3E+05	6.6E+05	6.5E+05	5.3E+05	7.2E+05	5.7E+05
Log reduction to inoculum			-1	-1	0	0	0	-1	0
Log reduction to 0h				0	0	0	0	0	0
Concentration 100 %	20 - 25	2.2E+05	3.8E+05	5.0E+05	6.1E+05	1.3E+06	6.8E+06	>30000000	>30000000
Log reduction to inoculum			0	0	0	-1	-1	<-2	<-2
Log reduction to 0h				0	0	-1	-1	<-2	<-2
Concentration 50 %	2 - 8	2.2E+05	6.3E+05	460000	5.0E+05	3.9E+05	4.3E+05	3.7E+05	5.8E+05
Log reduction to inoculum			0	0	0	0	0	0	0
Log reduction to 0h				0	0	0	0	0	0
Concentration 50 %	20 - 25	2.2E+05	4.3E+05	6.9E+05	8.4E+05	2.3E+06	1.2E+07	>30000000	>30000000
Log reduction to inoculum			0	0	-1	-1	-2	<-2	<-2
Log reduction to 0h				0	0	-1	-1	<-2	<-2
Positive Control	20 - 25	2.2E+05	4.3E+05	n.a	n.a	n.a	n.a	>30000000	>30000000

Abbreviations: Colony-forming unit (cfu).

https://doi.org/10.3390/bioengineering13040417
